# Effect of Digital Safety Interventions on Parental Practices in Safeguarding Children’s Digital Activities: Systematic Review and Meta-Analysis

**DOI:** 10.2196/70745

**Published:** 2025-10-10

**Authors:** Maggie Zgambo, Edah Anyango, Diana H Arabiat, Irene Ngune, Evalotte Mörelius, Min Zhang, Lisa Claire Whitehead

**Affiliations:** 1 School of Nursing and Midwifery Edith Cowan University Perth Australia; 2 ARC Centre of Excellence for the Digital Child Brisbane Australia; 3 Maternal and Child Nursing Department School of Nursing The University of Jordan Amman Jordan; 4 JBI The University of Jordan Health Research Centre University of Jordan Amman Jordan; 5 Department of Health, Medicine and Caring Sciences Linköping University Linköping Sweden; 6 The Centre for Evidence Informed Nursing, Midwifery and Healthcare Practice: A JBI Affiliated Group Adelaide Australia

**Keywords:** child, parental mediation, digital safety, parental education, meta-analysis, intervention effectiveness, screen time, digital technology, digital literacy

## Abstract

**Background:**

The prevalence of and growth in digital technology present opportunities for educational and social enrichment; however, there are also many health and digital safety risks for children engaging with digital technology. While there is a growing body of research on digital safety programs to enhance children’s digital safety through parental support, evidence regarding the effectiveness of such interventions remains limited.

**Objective:**

This study aimed to evaluate the effectiveness of digital safety interventions on parental practices related to safeguarding children’s digital activities.

**Methods:**

The review was conducted following the Joanna Briggs Institute methodology for systematic reviews and has been reported in accordance with the PRISMA (Preferred Reporting Items for Systematic Reviews and Meta-Analyses) guidelines. A comprehensive search was performed in May 2024 in MEDLINE, CINAHL Ultimate, PsycINFO, Web of Science, The Allied and Complementary Medicine Database, ProQuest Central, and IEEE Xplore databases to identify peer reviewed articles that were (1) published in English, (2) included parents as participants, and (3) reported on programs or interventions designed to enhance parents’ knowledge and skills to safeguard children’s digital safety.

**Results:**

A total of 11 published studies between 2012 and 2024 were included in the review. Data from 8 studies were included in the meta-analysis. A significant effect (Hedges *g*=–0.47; 95% CI –0.85 to –0.08; *P*<.001) was observed in children’s screen time in the 5 randomized controlled studies, while a large effect size was observed in 3 quasi-experimental studies (Hedges *g*=1.90; 95% CI –4.36 to 8.16; *P*<.001). A substantial level of heterogeneity was evident in the randomized studies (*I*^2^=87.95%). The quasi-experimental studies exhibited no heterogeneity (*I*^2^=0%). Overall, parents’ digital safety knowledge and skills improved as a result of the digital safety interventions. Notably, children’s screen time (*P*=.04) and parents’ own screen time (*P*=.001) decreased following the digital safety intervention.

**Conclusions:**

Parents who participate in digital safety interventions report higher levels of knowledge and skills related to digital safety, as well as a shift in attitudes, including the intention to implement strategies to safeguard their children engaging with technology. The enhanced knowledge and skills reported in these studies led to measurable reductions in both parents’ and children’s screen time. Future research should identify strategies that address community norms and other digital safety risks beyond screen time and bullying, which were predominant outcomes in these studies.

## Introduction

Technology has transformed the way children learn, socialize, and interact with the world. Internet and digital technology have made access to new information from afar more accessible and immediate [1,2]. Digital technology is embedded in the school system [3] and this means that children spend a significant portion of their time engaging with and using digital technologies to perform daily tasks such as homework, to form and nurture friendships, and for entertainment [1]. These digital advancements offer a wealth of opportunities for educational and social growth, with evidence indicating that moderate use of digital technology can positively impact children’s learning, development, and mental well-being [4,5], mainly through enhancing children’s literacy and numeracy skills, manual dexterity, and visuo-spatial working memory [6]. The rapid integration of digital technologies into daily life has increased the use of digital technology in schools and at home, with parents admitting being obligated to embrace the digital world that is often new to them, as their children now access web-based school learning resources, such as homework and timetables [7]. Digital technology use has impacted children of all ages, including children less than a year old, who spend time on digital media privately or independently [5]. However, in Australia, digital technology use and access to digital platforms increase with age, with adolescents (13-17 years) having access to various social media platforms, such as YouTube (86%), Facebook (75%), Snapchat (67%), and Instagram (70%) [8].

Healthy digital use among children has been debated, with Suresh and Tiwari [5] recommending minimal technology use of 1 to 2 hours a day for children aged 3 to 8 years. The American Academy of Pediatrics reinforces this stance, and they further recommend that children younger than 2 years should not engage in screen time [9]. In 2016, the Canadian Society of Exercise Physiology released the 24-hour movement guidelines tailored for children and young people aged 5 to 17 years, which are intended to assist parents and educators to promote appropriate daily levels of physical activity, manage screen time, and ensure adequate sleep duration [10]. Various countries, including Australia [11], South Africa [12], and New Zealand [13], along with international bodies such as the World Health Organization [14], have adopted these guidelines. However, experts from the Australian Research Council Centre of Excellence for the Digital Child have contended that while limiting screen time is often recommended in these guidelines, such restrictions may overlook potential developmental benefits [15].

Recent evidence indicates that children report spending up to 4 hours or more (outside their digital classes) of screen time on their technological devices, such as computers, television, smartphones, video games, and other mobile devices such as iPads [1]. Alongside its countless benefits, the digital space can pose several risks, particularly for children who lack the experience and understanding to navigate it safely. Scholars argue that the increasing use of digital technology among children has amplified their exposure to a broad spectrum of web-based risks, including cyberbullying, exposure to inappropriate content, privacy violations, digital addiction, and online sexual exploitation [16,17]. These threats can impact children’s mental well-being and lead to mental disorders, such as depression or anxiety [18,19]. Parents are tasked with the primary role of protecting their children’s well-being, and this now needs to encompass digital safety: defined in this review as safeguarding children from web-based risks such as cyberbullying, exposure to inappropriate content, privacy violations, online sexual exploitation and the inappropriate use of digital technologies including excessive screen time that can lead to digital addiction and negative mental health outcomes [20,21]. However, the digital landscape offers a high level of anonymity and accessibility that complicates direct oversight, making parents feel unequipped to navigate the vast and rapidly evolving digital space [22].

Therefore, it is essential to position parents as active participants in mitigating potential harm by developing effective digital safeguarding practices that facilitate children’s safety [23,24], giving parents a primary role in moderating the use of technology by monitoring, guiding, and supervising their children [5,24]. Banić and Orehovački [23] emphasize that parents must participate in their children’s digital technology usage. This means parents need to be equipped with the knowledge, skills, and values necessary to foster safe and responsible navigation of the digital landscape by children while safeguarding their well-being [1]. However, despite the wide embrace of digital platforms, the capacity of parents to effectively safeguard their children’s digital activities remains inconsistent, often constrained by varying levels of digital literacy, awareness of web-based risks, and access to appropriate resources.

Various digital safety interventions have been developed to equip parents with the knowledge and tools to protect their children in the digital space. Despite this growing number of digital safety interventions available to parents, there remains a lack of comprehensive evidence regarding their effectiveness in improving parental knowledge, skills, and practices and, ultimately, enhancing child safety on the web. Recent reviews have highlighted the significance of parental mediation through strategies such as educational programs, digital monitoring tools, parental controls, and strategies for fostering open communication between parents and children about web-based risks [23,25-27].

Existing reviews on the topic predominantly focused on the effects of digital literacy practices on children’s learning, identifying parental mediating strategies, and factors affecting parental mediation strategies [23,25-29]. None of the existing reviews has evaluated evidence on the effectiveness of the approaches in increasing the knowledge and skills of the parents, as well as enhancing the safe practices of using digital technology by their children. Aggregating evidence through a systematic review and meta-analysis has the potential to inform decision-makers by providing a comprehensive and critically evaluated synthesis of evidence while also uncovering research gaps. The rigorous statistical analysis involved in measuring the size of effect, examining the heterogeneity, and identifying possible sources of bias enhances the reliability of the synthesis. Therefore, this systematic review and meta-analysis evaluated the effectiveness of digital safety interventions in improving parental practices related to safeguarding children’s digital activities. The primary research question for this review was “What is the effectiveness of digital safety interventions on parental literacy and practices in increasing parental knowledge and skills on digital safety?”; the secondary review question was “What is the effectiveness of parental digital safety interventions on children’s digital safety?”

## Methods

### Design

The systematic review was conducted following the Joanna Briggs Institute (JBI) methodology for systematic reviews [30]. The review involved developing a clear and focused review question, establishing explicit inclusion and exclusion criteria, a systematic search of relevant databases, a critical appraisal of included studies, and a synthesis of findings. These steps adhered to the a priori protocol that was developed before commencing the study. The protocol was discussed by the members of the research team before being registered in the Open Science Framework [31].

### Eligibility Criteria

To be included in this review, peer reviewed articles published from database inception to 2024, were required to meet the following criteria: (1) population included parents or primary caregivers responsible for children aged 0-17 years; (2) irrespective of the delivery methods, interventions included resources, programs or interventions designed to enhance parents’ knowledge, and skills to safeguard children’s activities when interacting with digital technologies; (3) control groups received no intervention or were waitlisted for the intervention following the conclusion of the study; (4) primary or secondary outcomes on effectiveness of the intervention on parental practices and children’s digital safety were explicitly reported; and (5) interventional studies including randomized controlled trials (RCTs), non-RCTs, and quasi experiment. Articles were excluded if they were published in a language other than English and if they did not meet the inclusion criteria outlined above.

### Search Strategy

A 3-step search strategy was used in this review. The first phase of the search involved a limited exploratory search in 2 databases: MEDLINE (PubMed) and CINAHL (EBSCO). This preliminary search aimed to identify key articles that would inform subsequent search stages. The text words contained in the titles and abstracts of relevant articles and the index terms used to describe the articles were used to develop a comprehensive search strategy. The full search strategy was applied to the following databases: MEDLINE, CINAHL Ultimate, PsycINFO, Web of Science, The Allied and Complementary Medicine Database, ProQuest Central, and IEEE Xplore (Table 1).

**Table 1:** Search strategy.

Search 1	(Parent* OR famil*)
Search 2	((cybersafe* OR “cyber safe*” OR “digital* safe*” OR “digital security” OR “digital risk” OR “e safety” OR esafety OR “internet safe*” OR “internet security” OR “internet risk*” OR “online safe*” OR “online risk*” OR “online security” OR “safe technolog*”))
Search 3	(((MH “Child”) OR (MH “Child, Preschool”)) OR ((MH “Minors”)) OR ((MH “Adolescent”)) OR (child* OR minor* OR adolescen*))
Search 4	((“Parent* education” OR “Parent* knowledge” OR “Parent* support” OR “Parent* mediation” OR “famil* education” OR “famil* knowledge” OR “famil* support” OR “famil* mediation”))
Search 5	S1 AND S2 AND S3 (Parent* OR famil*)AND (cybersafe* OR “cyber safe*” OR “digital* safe*” OR “digital security” OR “digital risk” OR “e safety” OR esafety OR “internet safe*” OR “internet security” OR “internet risk*” OR “online safe*” OR “online risk*” OR “online security” OR “safe technolog*”) AND (((MH “Child”) OR (MH “Child, Preschool”)) OR ((MH “Minors”)) OR ((MH “Adolescent”)) OR (child* OR minor* OR adolescen*))
Search 6	S2 AND S3 AND S4 (cybersafe* OR “cyber safe*” OR “digital* safe*” OR “digital security” OR “digital risk” OR “e safety” OR esafety OR “internet safe*” OR “internet security” OR “internet risk*” OR “online safe*” OR “online risk*” OR “online security” OR “safe technolog*”) AND ((MH “Child”) OR (MH “Child, Preschool”)) OR ((MH “Minors”)) OR ((MH “Adolescent”)) OR (child* OR minor* OR adolescen*) AND ((“Parent* education” OR “Parent* knowledge” OR “Parent* support” OR “Parent* mediation” OR “famil* education” OR “famil* knowledge” OR “famil* support” OR “famil* mediation”))

The databases were searched systematically using the identified keywords and MeSH (Medical Subject Headings) terms. No date or language limitations were applied. The Boolean operator “OR” was used to combine similar search terms, while “AND” was applied when combining different search terms. Two reviewers searched each database independently and then compared findings for similarities or any divergences. Last, a hand search of the reference lists of included studies, along with the use of the Scite artificial-powered research tool, was conducted to identify additional articles. The database search was conducted between May and August 2024.

### Study Selection

Identified citations were uploaded into EndNote (version X9; Clarivate) Reference Management System and duplicates removed [32]. The remaining titles were imported into the JBI System for the Unified Management, Assessment, and Review of Information (JBI SUMARI) [33]. Titles were then screened independently by 2 reviewers. Titles and abstracts deemed eligible were retained for level 2 screening, where full texts were retrieved and assessed independently by 2 reviewers. Disagreements occurring between the 2 reviewers at both screening levels were resolved with a discussion in the presence of team members to reach a consensus.

### Assessment of Methodological Quality

In this review, eligible studies underwent a rigorous critical appraisal conducted by 2 independent reviewers. The evaluation of the RCTs was performed using the JBI checklist for randomized controlled studies, which has 13 appraisal questions to assess bias related to selection and allocation of participants, administration of the intervention, assessment / detection / measurement of the outcome, participant retention, and data analysis [34]. Nonrandomized studies were assessed by 7 questions in the checklist (Multimedia Appendix 1) for quasi-experimental studies, and these questions assess bias in the domains of temporal precedence, selection and allocation of participants, confounding factors, administration of intervention, assessment/detection/measurement of the outcome, retention, and statistical validity [35]. The response options to each question are “Yes” (scores 1), “No” (scores 0), or “Unclear” (scores 0). The total score was converted into a percentage, and based on previous research [36], each study was ranked according to 3 levels of bias: high (for scores between 0% and 49%), moderate (for scores between 50% and 79%), and low (for scores between 80% and 100%). The specific questions used to assess the methodological quality of the included studies are detailed in Tables 2 and 3, which highlight the criteria followed during the appraisal process. In instances where discrepancies arose between the 2 reviewers, resolutions were achieved through collaborative discussion or a third reviewer to reach a consensus.

**Table 2:** Critical appraisal of eligible randomized controlled trials.

		Roberto et al [37]	Sanders et al [38]	Adams et al [39]	Birken et al [40]	Lin et al [41]	Raj et al [42]	Percentage
**Appraisal questions**								
	Was true randomization used for the assignment of participants to treatment groups?	Yes	Yes	Yes	Yes	Yes	Yes	100
	Was allocation to treatment groups concealed?	No	Unclear	Unclear	No	Yes	Yes	33.3
	Were the treatment groups similar at the baseline?	Yes	Yes	Yes	Yes	Yes	Yes	100
	Were participants blind to treatment assignment?	Unclear	Unclear	Unclear	Unclear	Yes	Yes	33.3
	Were those delivering the treatment blind to treatment assignment?	No	No	Unclear	Yes	Yes	No	33.3
	Were outcome assessors blind to treatment assignment?	No	No	Unclear	Yes	Yes	Unclear	33.3
	Were treatment groups treated identically, other than the intervention of interest?	Yes	Yes	Yes	Yes	Yes	Yes	100
	Was follow-up complete and, if not, were differences between groups in terms of their follow-up adequately described and analyzed	Yes	Yes	Yes	Yes	Yes	Yes	100
	Were participants analyzed in the groups to which they were randomized?	Yes	Yes	Yes	Yes	Yes	Yes	100
	Were outcomes measured in the same way for treatment groups?	Yes	Yes	Yes	Yes	Yes	Yes	100
	Were outcomes measured in a reliable way?	Yes	Yes	Yes	Yes	Yes	Yes	100
	Was appropriate statistical analysis used?	Yes	Yes	Yes	Yes	Yes	Yes	100
	Was the trial design appropriate and any deviations from the standard randomized controlled trial design (individual randomization, parallel groups) accounted for in the conduct and analysis of the trial?	Yes	Yes	Yes	Yes	Yes	Yes	100
Percentage score per article		69	69	69	85	100	85	—^a^

^a^Not applicable.

**Table 3:** Critical appraisal of eligible quasi experiment.

		Uludaşdemir and Küçük [43]	Boonmun et al [44]	De Lepeleere et al [45]	Sadeghi et al [46]	Canpolat and Karadaş [47]	Percentage score per question
**Appraisal questions**							
	Is it clear in the study what is the “cause” and what is the “effect” (ie, there is no confusion about which variable comes first)?	Yes	Yes	Yes	Yes	Yes	100
	Were the participants included in any comparisons similar?	Yes	Yes	Yes	Yes	Yes	100
	Were the participants included in any comparisons receiving similar treatment/care, other than the exposure or intervention of interest?	Yes	Yes	Yes	Yes	Yes	100
	Was there a control group?	Yes	Yes	Yes	N	Yes	80
	Were there multiple measurements of the outcome both pre and post the intervention/exposure?	Yes	Yes	Yes	Yes	Yes	100
	Was follow up complete and if not, were differences between groups in terms of their follow-up adequately described and analyzed?	Yes	Yes	Yes	Yes	Yes	100
	Were the outcomes of participants included in any comparisons measured in the same way?	Unclear	Unclear	Unclear	Yes	Yes	100
Percentage score per article		100	100	100	85.7	100	—^a^

^a^Not applicable.

### Data Extraction

Data were independently extracted using the standardized JBI RCT data extraction forms by 2 independent reviewers and then crosschecked by a third reviewer. The extracted information included (1) attributes of the study: author, geographical location, year of publication, economic background, setting, study aims; (2) methodological qualities: study design, sample size/ population, characteristics of intervention, statistical analyses; and (3) findings and conclusions (Multimedia Appendix 2 [37-49]).

### Data Synthesis

Data synthesis was conducted through meta-analysis and narrative synthesis. Meta-analysis was conducted when studies demonstrated a sufficient degree of homogeneity in terms of participants, interventions, and outcomes. Based on these criteria, a meta-analysis was conducted specifically for the outcome of children’s screen time. Forest plots were generated to show mean differences with 95% CIs and funnel plots. Random-effects meta-analyses using the restricted maximum likelihood method were used to pool the data. Heterogeneity was assessed using an *I*^2^ test, with values <50% considered nonsubstantial. The outcome was deemed statistically significant if the *P* value was less than .05. Effect sizes were interpreted according to GRADE (Grading of Recommendations, Assessment, Development, and Evaluation) recommendations: small (<0.5), moderate (0.5-0.8), and large (> 0.8) effects [50]. Sensitivity analyses using the leave-one-out approach were performed to explore sources of heterogeneity. Subgroup analyses and meta-regression were not conducted due to the insufficient number of studies in both RCT and quasi-experimental meta-analyses. For other outcomes (eg, parental awareness, attitudes) with substantial conceptual heterogeneity measured by different instruments, a narrative synthesis was undertaken with supporting tables due to the inappropriateness of statistical pooling. Publication bias was evaluated through funnel plot visualization, Egger regression test, and trim-and-fill analysis. All analyses were conducted using Stata (version 17.0; StataCorp LLC).

## Results

### Study Selection

**Figure 1:**
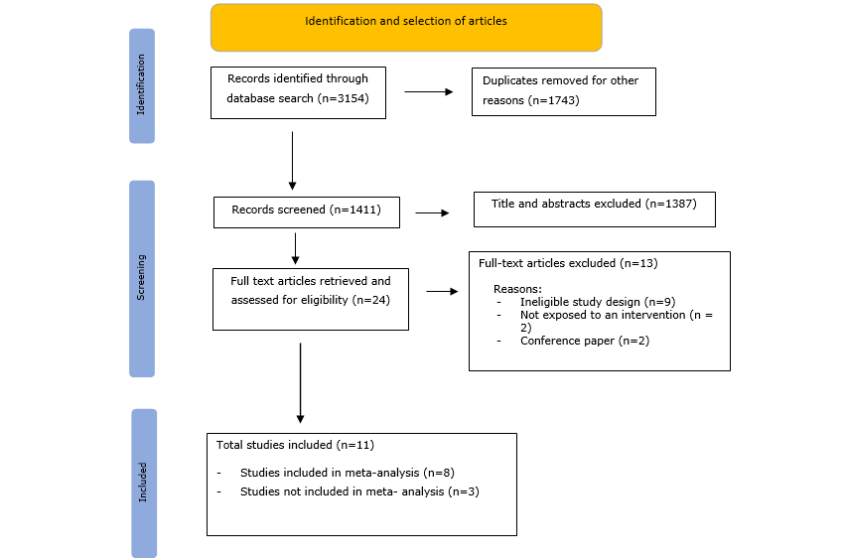
PRISMA (Preferred Reporting Items for Systematic Reviews and Meta-Analyses) flow diagram.

After removing duplicates from a total hit of 3154, a total of 24 potentially eligible titles and abstracts were retained from 1411 records. Following a full-text assessment of 24 articles, 13 were excluded based on the following exclusion criteria: ineligible study design (n=9), lack of intervention exposure (n=2), incompatible participant characteristics (n=1), ineligible outcomes (1), conference proceedings (n=2). Eleven studies were included in this systematic review and meta-analysis, and details are provided in Figure 1.

### Methodological Quality

Overall, the risk of bias score for RCTs was low to moderate (average score of 82%; range 69% to 100%; Table 2). Three articles scored moderate risk [37-39], and another 3 articles scored low risk [40-42]. Studies that had a moderate risk were either unclear in their description or did not (1) conceal allocation of intervention groups, (2) blind participants to intervention groups, (3) blind those delivering interventions, and (4) outcome assessors were not blind to intervention assignment [37-39]. All quasi-experimental studies scored 100% except one article, which scored 85% [46], representing the lowest risk of bias (Table 3).

### Characteristics of Included Studies

The review included 11 studies published between 2012 and 2024. Study designs consisted of RCTs (6/11, 54.5%) and quasi-experimental studies (5/11, 45.5%). Studies were conducted across 8 countries: Turkey (2/11, 18.2%), Iran (2/11, 18.2%), the United States (2/11, 18.2%), and one study each from Thailand, Belgium, Canada, Malaysia, and Taiwan (1/11 each, 9.1%). Sample sizes ranged from 28 to 360 parent-child dyads. Regarding intervention delivery formats, studies used face-to-face sessions (5/11, 45.5%), online/web-based delivery (4/11, 36.4%), and combined web-based and face-to-face delivery (2/11, 18.2%). Participants were primarily parents (with mothers comprising >80% in most studies) and children ranging from toddlers (18 months) to adolescents. The duration of interventions varied from a single session to 9 weeks. The follow-up periods ranged from immediate postintervention to 4 months. The contents of interventions mainly focused on 3 domains: cyberbullying prevention (3/11, 27.3%), screen time management (5/11, 45.4%), and gaming-related parenting practices (3/11, 27.3%). Detailed characteristics of the included studies are presented in Multimedia Appendix 2 [37-49].

### Summary of Effects of Digital Safety Interventions on Parental Outcomes

#### Parental Digital Safety Knowledge, Attitudes, and Skills

Six studies reported the effectiveness of digital safety interventions in improving parents’ digital safety knowledge and skills (Table 4) [37,42-45,47]. For cyberbullying-related outcomes, significant improvements were observed in both parental awareness [43] and various communication intentions [37] (all *P*<.05). Screen time management interventions showed mixed results: while parental attitudes demonstrated significant improvements (*P*<.05), changes in intentions and subjective norms remained nonsignificant [44]. Raj et al [42] in their larger study (n=360) found significant enhancements in parental knowledge and self-efficacy for screen time reduction (*P*<.001). However, De Lepeleere et al [45] found no significant improvements in parental self-efficacy for either modeling or permission concerning gaming (*P*>.05), suggesting limited intervention effectiveness in this domain. Overall, the interventions appeared most effective in enhancing cyberbullying-related competencies, moderately effective for screen time management, but showed minimal impact on gaming-related outcomes.

**Table 4:** Effects of digital safety interventions on parental digital safety knowledge and skills. Effect sizes and statistical tests are presented as reported in the original studies. For repeated measures, only postintervention and final time point results are shown.

Reference	Sample size (n/N)^a^	Outcome measure	Intervention results							Timing of measurement
			Effect estimate					*P* value		
			Z test^b^	*F* test (*df*)	*t* test (*df*)	Mean difference (95% CI)				
Uludaşdemir and Küçük [43]	33/31	Parent cyberbullying awareness form	−2.035	—^c^	—	—	.04		Postintervention (5-week)	
Boonmun et al [44]	35/35									
		Parents’ attitudes (children’s screen time reduction)								
			—	—	—	2.37 (0.45 to 4.29)	.015		Postintervention (2-week)	
			—	—	—	2.06 (–0.79 to 4.91)	.16		2-month follow-up	
		Parents’ perceived behavioral control								
			—	—	—	1.29 (–1.05 to 3.62)	.28		Postintervention (2-week)	
			—	—	—	2.63 (0.19 to 5.07)	.04		2-month follow-up	
		Parents’ intentions (children’s screen time reduction)								
			—	—	—	0.11 (–0.92 to 1.14)	.83		Postintervention (2-week)	
			—	—	—	0.25 (–0.89 to 1.39)	.67		2-month follow-up	
		Parents’ subjective norms (children’s screen time reduction)								
			—	—	—	–0.91 (–3.49 to 1.66)	.49		Postintervention (2-week)	
			—	—	—	–0.55 (–3.24 to 2.15)	.69		2-month follow-up	
Canpolat and Karadaş [47]	14/14	Digital parental awareness scale (risk protection)								
			—	31.709 (1,26)^d^	—	—	<.001		Postintervention	
			—	—	—	—	<.001		3-month follow-up	
Raj et al [42]	180/180									
		Parental knowledge	—	—	—	6.88 (6.11 to 7.65)	<.001		3-month follow-up	
		Parental perception	—	—	—	–0.86 (– 0.98 to −0.73)	<.001		3-month follow-up	
		Parental self-efficacy (screen time reduction)	—	—	—	1.59 (1.48 to 1.70)	<.001		3-month follow-up	
De Lepeleere et al [45]										
	44/70	Parental self-efficacy (gaming-modeling)	—	1.11 (1, 112)^d^	—	—	.29		1-month follow-up	
	50/65	Parental self-efficacy (gaming-permission)	—	0.78 (1, 112)^d^	—	—	.38		4-month follow-up	
Roberto et al [37]	26/25									
		Parents’ perceived susceptibility (child cyberbullying risk)	—	—	2.76 (49)	—	.01		Postintervention (one session)	
		Parents’ intentions to talk to their child about saving evidence if cyberbullied	—	—	2.19 (49)	—	.03		Postintervention (one session)	
		Parents’ intentions to talk to their child about not retaliating if cyberbullied	—	—	2.66 (49)	—	.01		Postintervention (one session)	
		Parents’ intentions to talk to their child about telling an adult if cyberbullied	—	—	2.24 (49)	—	.03		Postintervention (one session)	

^a^n/N: Sample size of treatment group/control group or pretest/posttest.

^b^Mann-Whitney U test.

^c^Not applicable.

^d^Degrees of freedom for *F* tests were calculated based on sample sizes (*df*_1_=1, *df*_2_=total sample size of 2).

#### Parents’ Behavioral Outcomes

Based on data from 6 studies [37,42-45,47], including 827 participants, collectively examining behavioral outcomes, digital safety interventions showed mixed results in changing parents’ behaviors (Table 5). Only 2 outcomes demonstrated significant improvements: parents’ immediate screen time reduction (for child) behaviors (*P*=.05) and parents’ own screen time at 3-month follow-up (*P=*.001) [42,44]. However, interventions showed no significant effects on cyberbullying incidents and victimization, television and gaming-related parenting practices (rules, modeling, monitoring, and explanations), or technology-specific parenting (all *P*>.05), suggesting limited effectiveness in achieving sustained behavioral changes (Table 5).

**Table 5:** Effects of digital safety interventions on parents’ behavioral outcomes.

First author	Sample Size (T/C)^a^	Outcome measure	Intervention results						Timing of measurement
			Effect estimate				*P* value		
			Z test^b^	Mean difference (95% CI)	*F* test (*df*)				
Uludaşdemir and Küçük [43]	33/31								
		Cyberbullying incidents - The adolescents’ RCBI-II^c^	0.096	—^d^	—	.92		Postintervention (5-week)	
		Cyber Victimization - The adolescents’ RCBI-II	−1.124	—	—	.26		Postintervention (5-week)	
Boonmun et al [44]	35/35	Parents’ behaviors concerning children’s screen time reduction questionnaire							
			—	3.46 (0.03 to 6.88)	—	.05		Postintervention (2-week)	
			—	1.64 (–1.65 to 4.92)	—	.33		2-month follow-up	
Raj et al [42]	180/180	Parents’ screen time	—	–70.43 (–91.51 to 49.35)	—	.001		3-month follow-up	
De Lepeleere et al [45]									
	41/66	Rules concerning television	—	—	0.00 (1, 105)^e^	.97		1-month follow-up	
	45/69	Modeling concerning television	—	—	1.79 (1, 112)^e^	.18		1-month follow-up	
	50/63	Modeling concerning television	—	—	1.57 (1, 111)^e^	.21		4-month follow-up	
	37/61	Rules concerning gaming	—	—	1.16 (1, 96)^e^	.28		1-month follow-up	
	45/69	Modeling concerning gaming	—	—	2.42 (1, 112)^e^	.12		1-month follow-up	
	37/45	Giving an explanation concerning television	—	—	0.1 (1, 80)^e^	.75		4-month follow-up	
	28/40	Giving an explanation concerning gaming	—	—	0.29 (1, 66)^e^	.59		4-month follow-up	
	40/54	Monitoring gaming	—	—	1.84 (1, 92)^e^	.17		4-month follow-up	
Sanders et al [38]	17/15	Technology-specific parenting	N/A^f^	N/A	N/A	.48		Postintervention (one session)	

^a^T/C: treatment/ control.

^b^Mann-Whitney *U* test.

^c^RCBI-II: Revised Cyber Bullying Inventory.

^d^Not applicable.

^e^Degrees of freedom for *F* tests were calculated based on sample sizes (*df*_1_=1, *df*_2_=total sample size of 2).

^f^N/A: Not available.

### Meta-Analysis Results

#### Effects of Digital Safety Interventions on Children’s Screen Time

**Figure 2:**
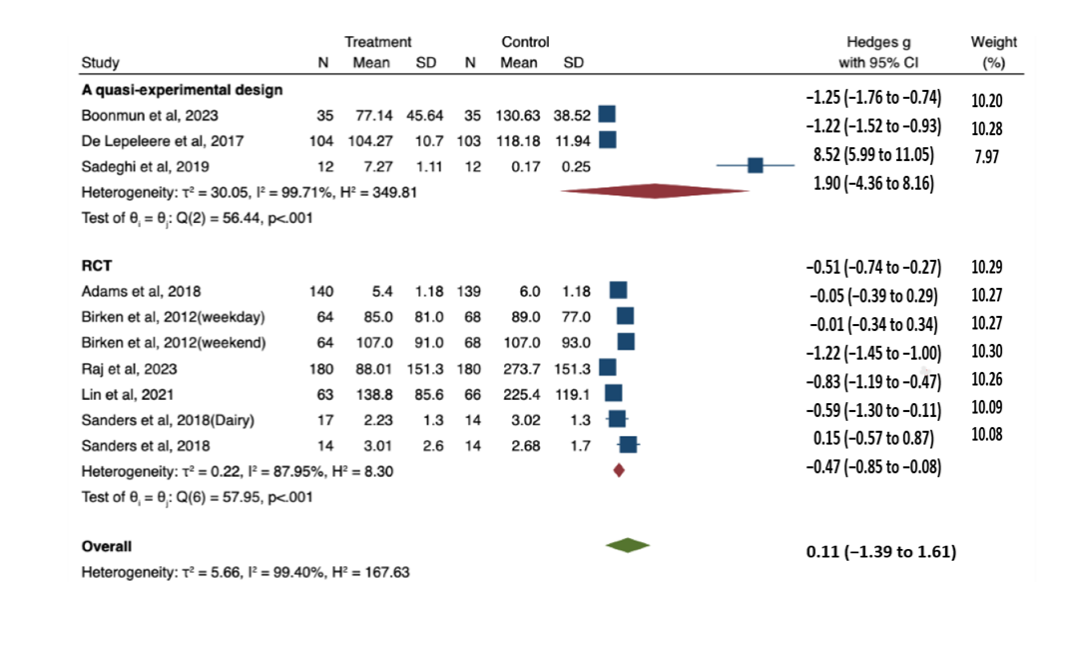
Meta-analysis of randomized controlled trials and quasi-experimental studies on the effects of digital safety interventions on children’s screen time [38,40-44,46,47]. REML: restricted maximum likelihood.

Figure 2 [38-42,44-46] presents a forest plot from a meta-analysis comparing treatment and control groups across 2 study types: quasi-experimental designs and RCTs. The quasi-experimental studies, comprising 3 studies and 151 participants, demonstrated a large effect (Hedges *g*=1.90; 95% CI –4.36 to 8.16; *P*<.001) with substantial heterogeneity (*I*^2^=99.71%). Conversely, the RCTs, comprising 5 studies (7 datasets) and 1091 participants, revealed a smaller yet significant effect (Hedges *g*=–0.47; 95% CI –0.85 to –0.08; *P*<.001) with substantial heterogeneity (*I*^2^=87.95%). The statistical analysis of group differences (Q=0.55, *P*=.46) indicates no significant disparities between the quasi-experimental and RCT designs.

#### Descriptive Comparison by Control Group Type and Delivery Mode

Among the 5 RCTs (7 datasets) included in the meta-analysis, formal subgroup analyses could not be conducted due to most subgroups having fewer than 2 studies. Therefore, effect size was compared descriptively by intervention delivery modes and control group types, with results interpreted cautiously given these limitations. Studies using waitlist controls [38,41,42] showed effect sizes ranging from –0.59 to –1.22, whereas studies with an active control [39] and standard care control [40] yielded effect sizes of –0.51 and –0.05, respectively. Regarding the mode of delivery, face-to-face interventions [38-41] showed variable effects (–0.05 to –0.83), while a single web-based intervention [42] demonstrated an effect size of –1.22 (Figure 2 [38-42,44-46]).

#### Sensitivity Analysis

**Figure 3:**
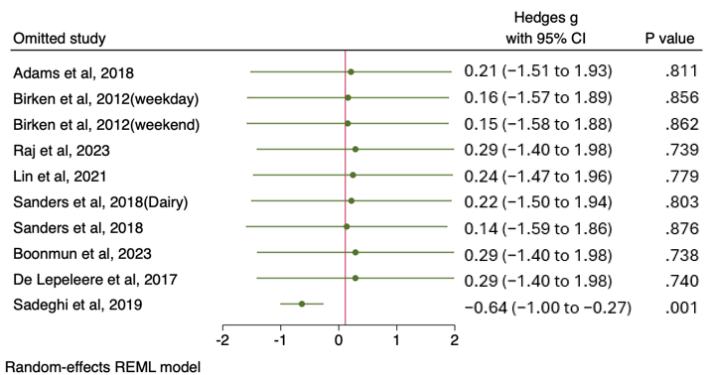
Leave-one-out sensitivity analysis on children’s screen time [38,40-44,46,47]. REML: restricted maximum likelihood.

The leave-one-out sensitivity analysis demonstrated the robustness of the meta-analysis findings for children’s screen time, with Hedges *g* values ranging from 0.14 to 0.29 and all 95% CIs crossing zero (*P*>.05), except when Sadeghi et al [46] was excluded (Hedges *g*=–0.64, *P=*.001) (Figure 3 [38-42,44-46]). The most pronounced effect was observed when the studies by Raj et al [42], Boonmun et al [44], Birken et al [40], or De Lepeleere et al [45] were excluded (Hedges *g*=0.29), while the smallest effect was noted when the study by Sander et al [38] or were omitted (Hedges *g*=0.14). These results indicate that the overall findings are not disproportionately influenced by most studies, except for Sadeghi et al [46], which appears to have a significant impact on the pooled effect estimate.

#### Publication Bias

**Figure 4:**
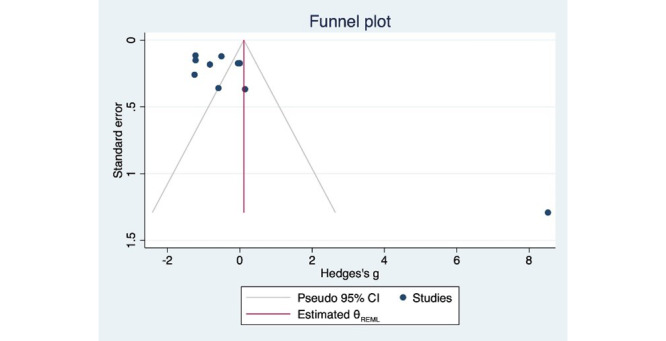
Funnel plot of the random-effects REML model. REML: restricted maximum likelihood.

**Figure 5:**
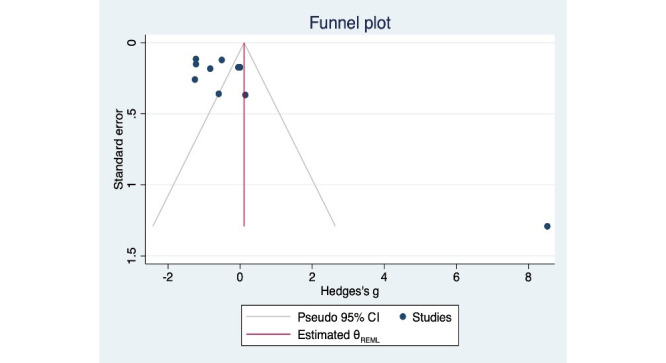
Funnel plot after trim-and-fill analysis. REML: restricted maximum likelihood.

Visual inspection of the funnel plot from the random-effects restricted maximum likelihood model (Figure 4) showed relative symmetry, and the Egger test indicated no significant publication bias (*t*=2.04, *P*=.08). The trim-and-fill analysis yielded an identical funnel plot (Figure 5) with zero missing studies, further confirming the absence of publication bias. The test of residual heterogeneity was significant (Q-res=83.64, *P*<.001). This suggests that while there is substantial heterogeneity in the included studies, there is no significant indication of publication bias in our meta-analysis.

## Discussion

### Principal Results

To the best of our knowledge, this is the first meta-analysis to assess the effectiveness of digital safety interventions on parental practices related to safeguarding children’s digital activities. This systematic review and meta-analysis identified 6 RCTs and 5 quasi-experimental studies to evaluate the impact of digital safety interventions on parental practices. Based on the comprehensive search of available studies, this area of research has been given limited attention. Nonetheless, the results provide evidence that parents who participate in digital safety interventions have a higher chance of increasing digital safety knowledge and skills, with a positive change in attitudes, including intentions to use strategies to safeguard their children engaging with technology.

Findings demonstrate that interventions targeting parents’ knowledge related to digital safety were more effective in intervention groups compared with control groups. While increased knowledge is associated with a behavior change [51,52], knowledge alone does not necessarily translate to a change in behavior [53,54]. Theories of Behavior Change [55] such as the Theory of Planned Behavior, emphasize that an individual’s likelihood of changing their behavior depends on their attitudes, social norms (which refer to beliefs about what others think regarding certain behaviors), and behavioral control (the individual’s ability to act) [56]. Similarly, the Social Cognitive Theory by Albert Bandura highlights personal (individual’s self-efficacy towards behavior), environmental (situational influences), and behavioral (having the ability to perform the behavior) factors as important elements in changing one’s behaviors [57]. Therefore, the effectiveness of an intervention such as those aimed at promoting parental support in safeguarding children engaging in digital technology relies not only on knowledge but also on enabling a shift in social norms and addressing contextual factors that influence behavioral control. While it is commendable that 7 of the 11 articles used either a model, a theory, or a framework to underpin their interventions, the inclusion of social norms or environmental influences in the targeting interventions remains insufficient. Future studies and interventions should consider strategies that can address community norms around the digital safety of children.

In this review, some included studies evaluated either knowledge or behavioral outcomes independently, and all studies used educational sessions as an intervention (or part of the interventions). These educational sessions were delivered through different approaches, including videos, infographics, presentations face to face, role play, in groups, or via web. It is, therefore, limiting to isolate the effect of an individual approach to parent education to influence their knowledge or behavior. Regarding the behavior outcomes, while the desired direction of effect is noted in most of the reported effect estimates, the majority were not statistically significant, except for 2 outcomes: parents’ own screen time [42] and children’s screen time [44], which reduced after exposure to digital interventions. Similar findings were reported in a previous meta-analysis, where the behaviors of youths did not change after exposure to anti-bullying interventions [58]. To further the understanding of the effectiveness of digital interventions, future research should consider evaluating the individual effects of components of the intervention. In addition, interventions predominantly targeted cyberbullying and screen time. Considering that digital safety encompasses broader outcomes beyond cyberbullying and screen time, we recommend that interventions targeting digital safety should adopt a holistic framework rather than addressing isolated components where applicable. There is a need for additional research that can measure digital safety outcomes that have not been included in these studies, albeit important, such as exposure to inappropriate content, web-based grooming or sexual exploitation, and digital citizenship.

Small effect sizes were also observed on giving an explanation concerning TV (*F*_1,80_=0.10; *P*=.75) or gaming (*F*_1,66_=0.29; *P*=.59), cyberbullying incidents (t/z=−1.124; *P*=.26), and rules concerning TV (*F*_1,105_=0.00; *P*=.97). The findings suggest an influence of the study design on the observed effect sizes as indicated in the significant difference between quasi-experimental studies and RCTs (Q=10.72, *P*<.001). The larger effect observed in the quasi-experiment could be because of the lack of randomization in these studies [59]. This potential bias, along with the detected substantial heterogeneity observed in RCTs, limits the reviewers’ ability to draw conclusions based on this finding. Therefore, further investigations, with an improved study design that involves implementors and outcome assessors, are required to understand the effect of digital interventions on children’s screen time and other studied outcomes.

Overall, the outcome of the analysis indicates that articles included in this review and meta-analysis were of high quality, with the majority (n=8/11) considered to have a low risk of bias. Another strength of the study lies in the systematic and robust search strategies used to identify the included studies. Just like other systematic reviews and meta-analyses, our study has some limitations. The search for articles did not extend to gray literature databases, which may have missed relevant studies conducted by reputable organizations and government agencies [60]. Results from the sensitivity analysis demonstrate a significant influence of one article [46] on children’s screen time despite a small sample size of 12; therefore, its validity should be interpreted with caution. Last, studies published in languages other than English were excluded due to time and financial constraints associated with translation, which may limit generalization of the outcomes.

### Conclusions

To the best of the reviewers’ knowledge, this is the first review to evaluate the effectiveness of digital safety interventions on parental practices related to safeguarding children’s digital activities. Several interventions were identified, with educational sessions being the most common intervention across the studies. The analysis for this study indicates that the identified interventions effectively increased parents’ knowledge and skills to safeguard their children while engaging with technology. Future research should use more robust designs that include blinding of outcome assessors and consider environmental factors. There is a need to focus on examining the effect of individual approaches to educational sessions on parents’ practices.

## Appendix

Multimedia Appendix 1 PRISMA 2020 checklist.

Multimedia Appendix 2 Characteristics of included studies.

## Abbreviations

GRADE: Grading of Recommendations, Assessment, Development, and Evaluation
JBI: Joanna Briggs Institute
MeSH: Medical Subject Headings
PRISMA: Preferred Reporting Items for Systematic Reviews and Meta-Analyses
RCT: randomized controlled trial
