# Supplementary material for: Effect of Digital Safety Interventions on Parental Practices in Safeguarding Children’s Digital Activities: Systematic Review and Meta-Analysis
Source: JMIR Pediatr Parent. 2025 Oct 10;8:e70745. doi: 10.2196/70745 (PMC12552826; doi:10.2196/70745)
Supplement: Multimedia Appendix 2 [file pediatrics_v8i1e70745_app2.docx]

Table S1. Characteristics of Included Studies.

| **Author and country** | | | | **Design/Setting** | | **Participant characteristics** | **Groups and intervention** | **Outcomes measured** | | **Description of main results** |
| --- | --- | --- | --- | --- | --- | --- | --- | --- | --- | --- |
| Roberto, Eden [39]  USA | | | | Randomised controlled study.  Middle school | | 51 parents of middle school children  84.3% female and 15.7% men  23.5% were aged < 35 years, 9.8% were aged between 36–40 years, 29.4% were aged from 41–45 years, 25.5% belonged to the 46–50 age bracket and 11.89% were 51years old or older  92% were the student's biological parents, 4% were stepparents, and 4% were another relative (i.e., older sister/brother, aunt/uncle, grandmother/grandfather, etc.).  Students gender: girls (66.7%) and boys (33.3%)  Education level of students: 5th grade (21.6%), 6th grade (25.5%), 7th grade (37.3%), or 8th grade (15.7%).  Race/ethnicity: white (76.5%), black (11.8%), Hispanic, (7.8%) and American Indian (3.9%). | An experimental group viewed the presentation before completing the survey measuring  A control group that completed the survey measuring the dependent variables before viewing the presentation  The intervention was an existing presentation by the Arizona Attorney General’s cyber-safety promotion presentation  45 and 55 minutes long  Focused on how perceived susceptibility to cyberbullying was addressed, defining cyberbullying, providing several anecdotal examples and news stories that demonstrated cyberbullying and the negative impacts of cyberbullying  The presentation concluded by reviewing ways parents could talk about cyberbullying with their children and strategies | Perceived susceptibility to cyberbullying  Behavioural intentions to talk to their children about saving evidence when cyberbullied Intentions to talk to their children about not retaliating when cyberbullied Intentions to talk to their children about telling a trusted adult when cyberbullied  An adapted survey was used [59, 60] | | There were Higher perceptions of susceptibility in the intervention group than the control group. The mean for experimental group 4.59 (SD=0.69), the mean for Control group 3.99 (SD=0.86) and t(49) = 2.76, p= 0.01, d= 0.79),  Greater behavioural intentions to talk to their children about saving evidence when cyberbullied in the experimental group t (49) = 2.19, p= 0.03, d = 0.63, the mean for experimental group 4.81 (SD= 0.63), and the mean for control Group 4.20 (SD=1.26)  There were higher intentions than the control group to talk to the child to not retaliate when cyberbullied t (49) = 2.66, p= 0.01, d = 0.76. the mean for experiment group 4.96 (SD= 0.20), and the mean for control group was 4.32 (SD= 1.22)  There were greater intentions to talk to children about telling a trusted adult in parents in the experiment group than control group t (49) = 2.24, p = 0.03, d = 0.64, the mean for experiment group 5.00 (SD=0.00), and the mean for control group mean 4.56 (SD= 1.00) |
| Aim: To evaluate the short-term effects of the Arizona Attorney General’s cyber-safety promotion presentation | | | | | | | | | | |
| Raj, Ahmad [43]  Malysia | | | | A single-blind, 2-arm cluster RCT  16 Government preschools and low-income communities | | 360 mother-child dyads.  The inclusion criteria for preschools were government preschools with classes for children aged 3 to 4 years.  Mothers with preschoolers aged 3 to 4 years, registered with government preschools.  Mothers reporting their children’s screen time as >1 hour per day in the past month.  Mother with access to smartphones and be willing to use WhatsApp as a medium of interaction.  Mothers with physical or mental disabilities and children with physical disabilities, as certified by medical practitioners, were excluded from the study.  Age: Preschool children aged 3 to 4 years.  Mother’s age, mean (SD): Intervention 34.1 (4.3), Control 33.4 (4.7). Female (%): Parents:100%  Child’s sex: 81+84/360  Race/Ethnicity: Malay: 166+174/360, non-Malay 20/360 | The intervention group comprised of 8 clusters with 360 mother-child dyads.  180 dyads were in the intervention group and another 180 in the waitlist control group.  The intervention was a 4-week "Stop and Play" intervention incorporated the 6 constructs of Social Cognitive Theory: knowledge, goal setting, self-efficacy, outcome expectation, observational learning, and problem-solving. It improved parental knowledge and skills  Culturally tailored activities were also provided as alternatives to screen time and promoted self-directed play as a potential solution to replace "digital babysitters".  The intervention module took 4 weeks, with approximately 60 minutes total time. It included weekly 3-minute videos, 2 infographics, and a 12-hour problem-solving session in the fourth week.  Mode of intervention delivery: WhatsApp application; YouTube video; Infographic materials; Private WhatsApp chat (problem-solving session)  Physical starter pack (including a "Stop and Play" fridge magnet and a screen media diary).  Who conduct the intervention: The delivery of the intervention module was performed by the primary researcher, a physician. Preschool educators distributed electronic pamphlets containing brief information about the study to all mothers. Eligible mothers who consented to participate were then added to a WhatsApp group managed by a research assistant.  Waitlist control group | The child's screen time was measured using a SCREENS questionnaire, covering television, computers, smartphones, and other mobile devices, calculated as average daily use.  Mother's screen time knowledge assessed with a 19-item questionnaire adapted from previous studies and WHO guidelines.  Mother's perception about screen time influence on child's well-being.  Mother's self-efficacy to reduce screen time and increase child's physical activity measured with 9 and 8 items respectively.  Mother's leisure screen time measured similarly to child’s  Physical household environment assessed by presence of screen devices in child's bedroom, creating a bedroom media score.  At the end of the study, the attrition rate was 1.7% | | Children in the intervention group were spending an average of 290.29 (SD 162.46) minutes on screen time per day as compared to an average of 273.69 (SD 139.04) minutes per day in the control group at baseline.  Increased knowledge and skills of children engaging in digital technology: Mothers in the intervention group showed significant improvements in knowledge (p<.001), attitudes (p<.001), self-efficacy (p<.001), and reduced their own leisure screen time (p<.001).  The intervention significantly reduced children's screen time by 202 minutes compared to the control group (β=-202.29, 95% CI -224.48 to -180.10; p<.001).  The intervention had no significant effect on the presence of digital devices in children's bedrooms.  Significant reduction in screen time was observed among children in the intervention group compared with those in the waitlist control group (F2,1068=173.07; P<.001). |
| Aim: To develop, implement, and evaluate the effectiveness of Stop and Play, a digital parental health education intervention to reduce excessive screen time among preschoolers from low socioeconomic families in Malaysia | | | | | | | | | | |
| Lin, Kuo [42]  Taiwan | | | | A clustered randomised controlled study with a parallel-group design  Fourteen private kindergartens | | 129 parent-child dyads: Intervention=63, Control=66  **Parents**  Aged ≥20 years, 36.8 ± 5.2 (range = 24–56) years.  Lived together with their children for at least 5 days/week and consented to participate.  **Children**  Similar teaching modes, a faculty–student ratio of 1:15, and government-accredited preschool programs.  The children were aged 4–6 years, were allowed a screen time of ≥2 h/day, and one of their parents was invited to participate in an educational intervention.  5.6 ± 0.7, aged 4–6 years  68 (52.7%) children were female.  Race/Ethnicity, n (%): Chinese (100%) | Intervention components: The 8-week parental educational program aimed to increase the parents' knowledge and self-efficacy with regards to screen use among children and the importance of monitoring and changing their children's screen behaviours.  Children in both groups attended their usual classes and carried out activities at their respective kindergartens.  This 8-week program was delivered for 50 min/week.  Courses were delivered through multiple strategies, such as reflection, group discussions, and role-play activities.  The text does not explicitly state who delivered the intervention.  The program was developed based on the theory of self-efficacy and reviewed by experts, but it does not specify who actually conducted the 8-week program with the parents.  Waitlist group | Children's screen time measured by parent-reported total screen time of their children, including watching television/DVD/videos, playing television games, or using a computer on weekdays and weekends.  Children's sleep quality and psychosocial adaptation: using the Children's Sleep Habits Questionnaire and the Paediatric Symptom Checklist-17. | | Effectiveness of digital safety interventions (Behavioural changes): For all children, the screen time was 211 ± 83.8 min.  After the intervention, the screen time of children in the experimental group was significantly reduced (effect size: 0.83, p < .001), and they presented improved sleep quality (effect size: 0.57, p= .01) and attention score (effect size: 0.77, p= .02) for psychosocial adaptation.  The experimental group showed significant decreases in overall, weekday, and weekend screen time (all p < .0001) after the intervention.  The control group showed no significant changes in screen time.  Significant group by time interactions were observed for all screen time measures (p < .01), indicating the intervention's effectiveness in reducing screen time compared to the control group.  The safety of children engaging in digital technology: The intervention significantly improved sleep quality (p=.02) and reduced attention problems (p=.03) in the experimental group while the control group showed no change in sleep quality and an increase in attention problems (p=.01).  Significant group by time interactions were observed for both sleep quality (p=.003) and attention problems (p=.004), but no significant differences were found in internalization and externalization scores between the groups.  Parental educational interventions potentially reduce preschoolers' screen time and improve their sleep quality and psychosocial adaptation. |
| Aim: To investigate the effect of a parental educational program on screen use, sleep quality, and psychosocial adaptation among preschoolers. | | | | | | | | | | |
| Adams, Marini [38]  USA | | | | A randomised controlled trial  Hershey Medical Centre | | Primiparous mother-newborn dyads (N = 279)  Intervention= 140, Control= 139  **Mothers**  Primiparous, English-speaking, and had a singleton infant born full-term (≥37 weeks gestation) with a birth weight ≥ 2500 g.  ≥20 years of age Intervention:28.7 ±4.6, Control: 28.7 ±4.9 years  Race/Ethnicity: Black 1; White 249; Native Hawaiian/Pacific Islander 1; Asian 9; Multi-racial 3  All mothers were married  Mothers’ education: high school level 32, some college 73, college and above 174. | The Intervention Nurses Start Infants Growing on Healthy Trajectories (INSIGHT) trial is a responsive parenting (RP) intervention  RP guidance provided to mothers included responding promptly, contingently, and in developmentally appropriate ways during different states of infant arousal, including fussing/crying, active/alert, drowsy, and sleepy.  The intervention promoted child self-regulation, emphasizing responsive feeding to encourage healthy food intake and weight, while also addressing sleep, emotional regulation, interactive play, and screen time.  The intervention was delivered at six time points: 3-4 weeks of infant age (home visit) 16 weeks of infant age (home visit) 28 weeks of infant age (home visit) 40 weeks of infant age (home visit) 1 year of infant age (research centre visit) 2 years of infant age (research centre visit)  Mode of intervention delivery: Face-to-face  Who conducted the intervention: Trained research nurses delivered the INSIGHT RP and control intervention material to mothers during one-on-one home visits  A safety control intervention: The control group received child safety messages that were matched for content intensity with the intervention group. These safety messages were centred on the same four infant behavioural states (drowsy, sleepy, fussy, and alert/calm) as the intervention group.  The content avoided any messages that could impact energy balance.  Topics included fire safety, prevention of falls, and toy safety. | Television exposure in the home; Television during meals; Daily screen time among children; Daily screen time among mothers; Enjoyment of tummy time at 8 weeks; Daily outdoor play at 2 years.  Screen time and television exposure were assessed at infants aged 44 weeks, and 1, 1.5, 2, and 2.5 years.  The interactive play was assessed at the child's age of 2 years. | | Daily screen time: RP mothers and RP children had less daily screen time than controls at each time point. (p ≤ 0.01).  Television during meals: Fewer RP than control group mothers reported the television was ever on during infant meals. (p < 0.05).  No screen time recommendation adherence: More RP than control parents reported their infants met the American Academy of Paediatrics’ no screen time recommendation at 44 weeks (53.0% vs. 30.2%) and at 1 year on weekdays (42.5% vs. 27.6%) and weekends (45.5% vs. 26.8%), but not after age 1 year.  Television exposure in the home: The television was on in the home on average fewer hours per day for the RP group compared to the control group (5.4 ± 0.1 vs. 6.0 ± 0.1 h/day, respectively). (p < 0.01).  Enjoyment of tummy time at 8 weeks: More RP infants enjoyed tummy time "most of the time" at 8 weeks of age, compared to control infants (51.3% vs. 33.6%, respectively). (p < 0.01)  Daily outdoor play at 2 years: More RP children used an outdoor play area daily, compared to children in the control group (30.0% vs. 15.1%). (p = 0.01) |
| Aim: To describe mothers and children’s screen time, television exposure, and interactive play from infancy to early childhood and to examine the effects of INSIGHT on mother’s and children’s screen time, television exposure, and interactive play. | | | | | | | | | | |
| Birken, Maguire [41]  Canada | | | | A randomised controlled trial  Community-based, primary care paediatric group practice | | 132 families: Three-year-old children and their parents  Intervention=64, Control=68  **Mothers**  Mothers born in Canada were 66% in the intervention group and 63% in the control group  Mother completed university degree were 81% in intervention group and 87% in control group  Mothers employed were 81% in intervention group and 78% in control group  **Children**  Attending day care or preschool: Intervention:67%, Control:78%  Age of children: Intervention: 3.12± 0.19, Control: 3.08 ±0.123  Female child: Intervention 56%, Control 51% | A short behavioural counselling intervention  Parents in the intervention group received a 10-minute behavioural counselling intervention by trained study personnel directly after the health maintenance visit.  A 10-minute, one-time intervention with1-year follow-up; Face-to-face  The intervention included information on the health impact of screen time in children and provided strategies to decrease screen time.  The strategies included removing the television from the child’s bedroom, encouraging meals to be eaten without the television on, and budgeting the child’s screen time.  Families were encouraged to try a 1- week television turn-off. Children were encouraged to spend time without television and were provided with a calendar and stickers to reward them for days without it.  Contingency planning for time spent not watching television was promoted.  Child activities during this session included stories to parents about television viewing (The Berenstain Bears and Too Much television) and creating a list of not television-related activities.  The intervention group also received a Canadian Paediatric Society handout titled “Promoting Good Television Habits"  Parents of children in both the intervention and control groups received standardized counselling on safe media use, which included information on television rating systems, Internet safety, and limiting exposure to violent programming.  Both groups received a previously published Canadian Paediatric Society parent handout titled “Managing Media in the Home.”  Intervention delivery: Study personnel graduate-level training in dietetics | The primary outcome was parent-reported screen time Secondary outcomes included television in the child’s bedroom, number of meals in front of the television, and Body Mass Index | | In the intention-to-treat analysis at 1 year, there were no significant differences in mean total weekday minutes of screen time (60, interquartile range [IQR]: 35–120 vs 65, IQR: 35–120; P = .68) or mean total weekend day minutes of screen time (80, IQR: 45–130 vs 90, IQR: 60–120; P = .33) between the intervention and control group. Adjusting for baseline BMI, there was a reduction in the number of weekday meals in front of the television (1.6 ± 1.0 vs 1.9 ± 1.2; P = .03) but no differences in BMI or number of televisions in the bedroom. Summary: This pragmatic trial of a brief intervention in the primary care setting was not effective in reducing screen time or BMI in 3-year-old children. However, there was a statistically significant reduction in the number of weekday meals in front of the screen. |
| Aim: To determine if an intervention for preschool-aged children in primary care is effective in reducing screen time, meals in front of the television, and BMI | | | | | | | | | | |
| Canpolat and Karadaş [48]  Turkey | | A quasi-experimental study  Two schools in the province of Malatya | | | | Overall, 40 parents: 23 females and 17 males  28 parents: Intervention =14, Control=14  Had to have children in secondary school  Age: Parents: Intervention 42.79 (5.56); Control 42.57 (6.54).  8 of the parents aged between 30 and 53 (M=40.30; SD=5.35) had one child, 20 had two and 12 had three children, and all of them had internet access at home  15 were mothers and 13 were fathers  Mother:15/28, Farther: 13/28 their children were in secondary school: 5th to 8th grade (100%) | Intervention components: A nine-week (session) Digital Parenting  Education Program: consisted of nine sessions covering digital world risks, digital parenting roles, being a digital role model, safe internet use software, social media security settings, digital games and risks, cyberbullying, technology addiction, digital footprints, and online rights.  Each session included discussions, demonstrations, and practical exercises to enhance parents' digital awareness and skills.  The intervention was conducted weekly for nine weeks, with each session lasting 90 minutes, with a follow-up measurement three months after the end of the training program.  Face-to-face delivery in-person group sessions.  Researchers participated in the education.  Waitlist group was used for control | Risk Protection sub-dimension of the Digital Parental Awareness Scale | | The safety of children engaging in digital technology: The experimental group's mean scores for "protection from risks" increased from pre-test (M=9.21) to post-test (M=14.50) and remained high at follow-up (M=14.43).  There was no significant difference between the pretest, posttest, and follow-up test scores of the control group participants.  Statistical analyses showed significant differences between the experimental and control groups, across measurement times, and in the interaction between group and time.  For the experimental group, significant differences were found between pre-test and both post-test and follow-up scores.  The Digital Parenthood Training Program significantly improved parents' digital parenting awareness levels in the "protection from risks" dimension for the experimental group, and this improvement was maintained three months after the intervention.  Conversely, the digital parenting awareness levels of the parents who did not participate in the digital parenting education program did not change significantly in the measurement made at the end of the application and afterwards. |
| Aim: To identify parents’ digital education needs, develop a functional training program to meet those needs, and test the effectiveness of the developed program | | | | | | | | | | |
| Boonmun, Rutja [46]  Thailand | | A quasi-experimental with non-randomised designs  Two childcare centres in a province in northeast Thailand | | | | 67 parents-child dyads: Intervention= 35 pairs, Control= 32 pairs.  **Parents**  Had preschool children who exhibited screen time of more than one hour per day; they were living together in the same household, had primary responsibility for their child’s care; and they could read and write Thai.  Parents' average age: Control:27.4 years, Intervention: 29.9 years.  Most parent participants were mother (number not provided)  **Children**  Children were aged 2-5 years with screen time of more than one hour per day. Children’s average ages: Control: 3.4 years, Intervention3.7 years | Sessions 1 and 2 followed Ajzen’s Theory of Planned Behaviour (attitudes, subjective norms, perceived behavioural control, and intentions), incorporating various activities and discussions to address each component in relation to children's screen time.  Session 3 focuses on parent-child interaction and continued to address components of the Theory of Planned Behaviour.  Post-test 1: parents answered all outcomes except parents’ behaviour and children’s screening time immediately after completing the program.  One week after completing the program, they answered behaviours and children’s screen time.  Post-test 2: 2-month after completing the program. A 2-week program: Week 1: Sessions 1-2: instructive and restrictive sessions (3 hrs; Week 2  Sessions 3: Parent-child interaction (3 hrs), face-to-face group discussions, role paly.  The experimental group received a 2-week program provided by the principal investigator.  The control group received a child’s screen time handbook after completing the data collection. | Television exposure in the home; Television during meals; Daily screen time among children; Daily screen time among mothers; Enjoyment of tummy time at 8 weeks; Daily outdoor play at 2 years.  Screen time and television exposure were assessed at infants aged 44 weeks, and 1, 1.5, 2, and 2.5 years.  The interactive play was assessed at the child's age of 2 years.  Used six questionnaires developed by the principal investigator | | Effectiveness of digital safety interventions (Behavioural changes): The findings of this study showed children’s screen time in the experimental group significantly differed from those in the control group (p < 0.05).  Children in the experimental group exhibited lower screen time than those in the control group at post-tests 1 and 2.  Children’s screen time in the practical decreased from 127.14 minutes/day to 70.64 minutes/day at post-test 1 and 77.14 minutes/day at post-test  The mean scores of parents’ attitudes and behaviours toward children’s screen time in the experimental group were significantly higher than those in the control group at post-test 1.  Increased knowledge and skills of children engaging in digital technology: The mean scores of parents’ perceived behavioural control on children’s screen time reduction in the experimental group were significantly higher than the mean scores in the control group at post-test 2. Conversely, the mean scores of parents’ norms and intentions in the experimental group were not significantly higher than those in the control group at post-test 1 and 2. |
| Aim: To examine the effects of the newly developed Parents’ Screen Time Reduction for Preschool Children Program, a training program for parents’ planned behaviours and the screen time reduction of their children | | | | | | | | | | |
| De Lepeleere, De Bourdeaudhuij [47]  Belgium | | A two-armed, quasi-experimental design  36 primary schools in Flanders (i.e., the Dutch-speaking part of Belgium) | | | | 207 parents: Intervention=104, Control=103  **Parents**  Had at least one primary schoolchild, availability of internet access at home, the primary school child is not on a diet nor has a physical disability.  Age of parent: Intervention: 40.5± 4.7, Control: 39.9 ±5.3.  Female parent: 92+90/207  **Children**  Age of child: Intervention: 9.2 ±1.5, Control: 9.6 ±1.6.  Female child: 47+53/207  Primary school children: 6- to 12-year-old children | 22 online videos about difficult parenting situations: These videos (5 on physical activity, 5 on screen time, and 12 on healthy diet (water, breakfast, fruit, vegetables, and buying healthy food in the supermarket)) each lasted about 2 min.  Video structure: Shows a difficult child-parent situation, Followed by an appropriate reaction of the parent. A narrator explains the parenting practices used in the video. 2 min* 22 per session over 4 weeks.  Week 1: 6 videos (3 on drinking water, 3 on eating fruit).  Week 2: Added 6 more videos (3 on eating vegetables, 1 on having breakfast, 2 on supermarket shopping).  Week 3: Added 5 videos on Physical Activity (PA).  Week 4: Added final 5 videos on screen-time. Web-based intervention: online videos and online questionnaire  Waitlist control group received no additional input during the period of the intervention, but got access to the online videos at the end of the study | Demographic variables, parent-reported child’s physical activity, screen-time and healthy diet (primary outcome), specific parenting practices (secondary outcome) and parental self-efficacy concerning these practices (secondary outcome) were assessed at baseline (T0), at one (T1) and at four (T2) months post baseline.  Used online questionnaire  Flemish Physical Activity Questionnaire and Food Frequency Questionnaire was used to assess child behaviour.  Girls Health Enrichment Multisite Study questionnaire assess parental self-efficacy | | Effectiveness of digital safety interventions (Behavioural changes): The intervention had no effect on children’s health behaviours reported by parents between baseline and 1-month follow-up (F = 0.15; p = 0.99) and between baseline and 4-months follow-up (F = 0.79; p = 0.59).  Increased knowledge and skills of children engaging in digital technology：Intervention effects on parental self-efficacy related to parenting practices: The intervention showed mixed effects on parental self-efficacy. At 1-month follow-up, there was a borderline significant increase in parents' self-efficacy for personal physical activity.  Intervention effects on parental self-efficacy were larger for parents of younger children (6–9 years old). |
| Aim: To evaluate the effect of ‘Movie Models’ on the child’s behaviour (PA, screen-time, healthy diet), parenting practices and parental self-efficacy | | | | | | | | | | |
| Uludaşdemir and Küçük [45]  Turkiye | | Study design A quasi-experimental study  In Anatolian high schools, in two districts of Ankara, in a city in the Central Anatolian region. Theoretical framework: The Health Promotion Model (HPM) developed by Pender (2011) | | | | 64 adolescents (intervention group = 33, control group = 31) and 64 parents (intervention group = 33, control group = 31)  The inclusion criteria for the adolescents included a score of 11 or above in the “I have engaged in cyberbullying” section of the RCBI-II and free access to the Internet, while the inclusion criterion for the parents was having a child who scored 11 or above in the “I have engaged in cyberbullying” section of the RCBI-II.  Those who did not complete the pre- and post-tests, and those who did not watch the learning videos, were excluded from the study  Age：57.6% of the adolescents in the intervention group and 51.6% in the control group were 15 years old; 51.5% of the parents in the intervention group and 51.6% of the parents in the control group were in the 40–45-year age.  In the intervention group, 78% of the adolescents were female, compared to 57.7% in the control group  There were 81.8% mothers in the intervention group compared to 83.9% in the control group.  Education level of students, n (%): 9th- and 10th-grade students, 100%; 63.6% of the adolescents in the intervention group and 64.5% in the control group were 9th-grade students. | Intervention components: The adolescents and parents took part in a 5-week web-based CBAE program and included educational videos on (1) the Definition and Types of Cyberbullying, (2) the Causes and Outcomes of Cyberbullying, (3) Digital Citizenship, (4) Empathy (5) and Safe Internet Use.  While the parents in the intervention group attended courses on (1) the Definition and Types of Cyberbullying, (2) the Causes and Outcomes of Cyberbullying, (3) Digital Parenting, (4) Empathy (5) and Safe Internet Use.  The web-based CBAE program materials comprised 10 educational videos – with five videos prepared each for adolescent and parent groups.  The research study lasted for 5 weeks; a one-week interval was provided between each video to ensure that each video was watched at least once.  Online through the website, SMS notification for any details related to intervention quality control, remind participants to watch the video  The Admin Panel provided the authors with statistics on how many times each user watched which video.  The Admin Panel was accessed daily, and a reminder was sent to participants who had not watched the video, encouraging them to watch it.  The researchers presented the educational material in the videos following the prepared script and using conversational language and easy-to-understand terminology, and the footage was recorded.  Waitlist group: No intervention | The Adolescent and Parent Data Collection Form, the RCBI-II and the Adolescent and Parent Cyberbullying Awareness Form.  The website garnered such statistical data as the frequency of logins by adolescents and their parents, the login days and times, and the duration of logins. | | Effectiveness of digital safety interventions: The RCBI-II: The CBAE program significantly reduced cyberbullying behaviours in the intervention group (Z = -3.240; p = 0.001; effect size = 0.6), while no significant change was observed in the control group (z = -1.761; p = 0.078; effect size = 0.2).  Neither the intervention nor control group showed significant changes in cyber victimization scores after the program.  Increased knowledge and skills of children engaging in digital technology: Significant improvements in both groups, with the intervention group demonstrating a larger increase (12.48 points, z = -4.675; p = 0.000) compared to the control group (z = -2.052; p = 0.040).  The between-group difference was statistically significant post-intervention (z = -1.929; p = 0.054), with the intervention group showing a higher effect size (1.352) than the control group (0.451).  The safety of children engaging in digital technology (Internet): The intervention group showed a statistically significant change in scores from baseline (11.21 ± 4.15) to post-intervention (14.37 ± 2.56) (z = -3.422, p = 0.001).  The control group's change from baseline (11.53 ± 4.17) to post-intervention (12.17 ± 4.81) was not statistically significant (z = -0.061, p = 0.951).  There was no statistically significant difference between the two groups at baseline (z = -0.534, p = 0.594). After the intervention, although the intervention group's mean score was higher than the control groups, this difference was not statistically significant (z = -1.686, p = 0.092). |
| Aim: To examine the efficacy of a web-based Cyberbullying Awareness and Education program that was designed to raise awareness of cyberbullying among adolescents and their parents. | | | | | | | | | | |
| Sadeghi, Pouretemad [44] | | A quasi-experimental design.  Tehran Autism Centre | | | | A total of 12 parents with children with subclinical autism symptoms aged 2–4 years  40 mother-young children: Internet-based intervention (n = 20 parent/child pairs); face-to-face intervention (n = 20 parent/child pairs)  **Children**  12 children with subthreshold autism symptoms who had not received an autism diagnosis aged 2 to 4 years  Exposed to digital devices for more than half their waking hours  Mean age of children: Intervention: 33.33, SD (9.95) month; Control: 31.90, SD (7.90).  **Parents**  Familiar with Skype software and video conferencing.  Age of mothers: Intervention: 35.50.45, SD (1.88) years; Control: 32.40, SD (2.91).  Age of fathers: Intervention: 32.17, SD (4.52) year; Control: 34.90, SD (3.24). | Intervention was based on Focused Playtime Intervention  Intervention components: Parents only attended both online and face-to-face sessions without their children. They received the parent training intervention (  All parents are trained to minimize their child’s screen time and to have interactions with children.  The intervention had of 8 parent training sessions (one session per week for 2-month, 90 min per group session and 30 min per individually session) aimed to increase the time parents and children spent; encourage the child to engage in communication with people rather than focusing on objects and to prevent solitary and repetitive.  Arousing a child to communicate with people and not objects  Preventing loneliness and repetitive activities  Removal of digital devices that interfere with parent-child interaction  Control: Face-to-face parent training intervention: Parent attendance to the face-to-face sessions was recorded weekly. Parents in both groups received intervention in the same treatment modules, chronological order and timeframe. | Screen time: using a parent-reports and Lifestyle checklist, recording children's activities every 5 minutes over two days.  Repetitive behaviours: using the Repetitive Behaviour Scale-Revised (RBS-R), a 43-item scale measuring six dimensions of repetitive behaviour.  EEG recording  Children were assessed by two experts with Ph.D. in child clinical psychologists and a child and adolescent psychiatrist  Screen time was determined by parents’ reports | | After intervention, the children's screen time significantly decreased from 7.27 h (SD, 1.11) in pretest to 0.17 h (SD, 0.25) in post-test and 0.29 h (SD, 40) in follow-up (F = 422.21, p < 0.0001, η 2 = 0.97, Power = 1).  The child was less likely to engage in media use |
| To examine the feasibility of recruiting, retaining, and implementing with fidelity and therapist competence of a one-session intervention for parents to reduce screen time; examine the feasibility of parents collecting daily diary screen time for a week and compare this method to parent appraisal of daily screen time, the standard in the field; examine preliminary data on changes in technology-specific parenting and child screen time; and examine parent satisfaction with the workshop and their sense of confidence in utilizing skills learned in the workshop to reduce child screen time. | | | | | | | | | | |
| Sanders et al. (2018) [40] | | A Randomised Controlled Trial: Community-based. Workshops were held at a university. | | 39 mother-young children’s dyads: Internet-based intervention (n = 20 parent/child dyads); face-to-face intervention (n = 20 parent/child dyads)  Children were aged 5 to 12 years. | | Intervention components: three one-hour hands-on workshops on Monday, Wednesday and Friday  First, the workshop to psychoeducation parents on the positive and negative effects of children's media use  Second, parenting skills were discussed as they pertained to technology-specific parenting strategies, including setting consistent boundaries around media use, providing effective instructions, and positive reinforcement.  Finally, parents were provided with specific information about setting parental controls and passwords for multiple categories of devices, including video game consoles, smartphones/tablets, and computers.  Parents were also given tips for various challenges in parenting that may occur for each of these devices (e.g., sharing device use among children) | Fidelity and Therapist Competence  Fidelity and Leader Competence  Parental appraisal of daily screen time  Parents were asked to report how many hours or minutes the child engages with technology  Dairy diary by parents  Parent Satisfaction and Confidence in Skill Implementation | The daily diary total screen time scores correlated with the estimated screen time scores at the baseline (r (31) = .57, p < .001) and post (r (28) = .68, p < .001) assessments  Parent reported daily diary screen time did not change from pre- to post-treatment for either group  The intervention group had a larger effect than the WL group for technology-specific parenting and parent appraisal of youth screen time  Two measures of screen time yielded substantially different means, particularly in the intervention group (daily screen time = 2.37 hours; appraised screen time = 4.36 hours) in terms of competence, parents believed they had the tools to implement controls for child screen time (item 7), and 95% of them had done so or were likely to do so (item 8) | |  |

Note: RCBI-II = The adolescents' Revised Cyber Bullying Inventory; CBAE=web-based Cyberbullying Awareness and Education; WHO= World Health Organisation
